# The economic burden of endoscopic treatment for anastomotic leaks following oncological Ivor Lewis esophagectomy

**DOI:** 10.1371/journal.pone.0221406

**Published:** 2019-08-28

**Authors:** Christoph Baltin, Florian Kron, Alexander Urbanski, Thomas Zander, Anna Kron, Felix Berlth, Robert Kleinert, Michael Hallek, Arnulf Heinrich Hoelscher, Seung-Hun Chon

**Affiliations:** 1 Department of Orthopedics and Trauma Surgery, University Hospital of Cologne, Cologne, Germany; 2 FOM University of Applied Sciences, Essen, Germany; 3 Department of Internal Medicine Med I, University Hospital of Cologne, Cologne, Germany; 4 Department of General, Visceral and Cancer Surgery, University Hospital of Cologne, Cologne, Germany; 5 Department of General, Visceral and Transplant Surgery, University Medical Center of the Johannes Gutenberg University, Mainz, Germany; 6 Center for Esophageal and Gastric Surgery, AGAPLESION Markus Krankenhaus, Frankfurt, Germany; Medical University Graz, AUSTRIA

## Abstract

**Background:**

Complications after surgery for esophageal cancer are associated with significant resource utilization. The aim of this study was to analyze the economic burden of two frequently used endoscopic treatments for anastomotic leak management after esophageal surgery: Treatment with a Self-expanding Metal Stent (SEMS) and Endoscopic Vacuum Therapy (EVT).

**Materials and methods:**

Between January 2012 and December 2016, we identified 60 German-Diagnosis Related Group (G-DRG) cases of patients who received a SEMS and / or EVT for esophageal anastomotic leaks. Direct costs per case were analyzed according to the Institute for Remuneration System in Hospitals (InEK) cost-accounting approach by comparing DRG payments on the case level, including all extra fees per DRG catalogue.

**Results:**

In total, 60 DRG cases were identified. Of these, 15 patients were excluded because they received a combination of SEMS and EVT. Another 6 cases could not be included due to incomplete DRG data. Finally, N = 39 DRG cases were analyzed from a profit-center perspective. A further analysis of the most frequent DRG code -G03- including InEK cost accounting, revealed almost twice the deficit for the EVT group (N = 13 cases, € - 9.282 per average case) compared to that for the SEMS group (N = 9 cases, € - 5.156 per average case).

**Conclusion:**

Endoscopic treatments with SEMS and EVT for anastomotic leaks following oncological Ivor Lewis esophagectomies are not cost-efficient for German hospitals. Due to longer hospitalization and insufficient reimbursements, EVT is twice as costly as SEMS treatment. An adequate DRG cost compensation is needed for SEMS and EVT.

## Introduction

In 2017, prognostic healthcare expenditures marked approximately € 1 billon per day in Germany (€ 374,2 billion in 2017) [1]. In the healthcare sector, inpatient treatment in hospitals is the leading cost driver. As Hospital costs make up about one third of all expenditures covered by public health insurances (€ 74,14 billion in 2017), total healthcare expenditures by public insurances are € 225.7 billion [2, 3].

A recent profit-center analysis of the revenues after esophagectomy has shown that a positive contribution margin can only be generated after uncomplicated esophagectomy [4, 5]. In a single center analaysis an uncomplicated course (Clavien-Dindo) after esophagectomy yielded a slightly positive profit margin of approximately € 2,500 [4]. Anastomotic leaks after esophageal surgery are one of the most severe and life-threating complications [6–8]. The risk for anastomotic leaks after esophageal cancer is significant as cohorts of high-volume centers worldwide show leak rates between 15.9% and 21.2% [1–3]. A common therapeutic endoscopic treatment of an anastomotic leak besides reoperation or conservative treatment is the implantation of a self-expanding metal stent (SEMS), a treatment with a success rate ranging from 69.3% to 91% [8, 9]. In recent years, endoscopic vacuum treatment (EVT) has become a promising new alternative for the therapeutic endoscopic treatment of anastomotic leaks as it has a sealing success rate of up to 90% [10]. The underlying indications for the SEMS and EVT method used for treatment were comparable and the endoscopist and surgeon could choose which method should be used.

However, in terms of the methods’ medical success, there is no conclusive evidence to suggest that one of these options (SEMS, EVT) is superior in the treatment of esophageal anastomotic leaks [9, 10]. Consequently, analyzing the cost-effectiveness of SEMS and EVT becomes a relevant means of determining a preferred treatment option. Therefore, the aim of our study was to analyze the economic burden, major cost drivers, and financial risks of endoscopic treatment of anastomotic leaks with SEMS and EVT after oncological surgery for esophageal cancer.

## Materials and methods

### Patient Group

During a 5-year period between January 2012 and December 2016, a total of 831 oncological esophagectomies were performed at the high-volume center of the Department of General, Visceral and Tumor Surgery of the University of Cologne (Chairman Prof. Dr. med. A.H. Hoelscher until 04 / 2016). All surgical procedures were esophagectomies with an intrathoracic anastomosis performed with curative intent. In this study, we include all patients who received a SEMS, EVT, or both for esophageal intrathoracic anastomotic leaks after Ivor Lewis esophagectomy during this period. Data was collected retrospectively from our endoscopic database “Clinic WinData” (version 8.05; E&L medical system GmbH, Erlangen, Germany) and from our hospital database “Orbis” (version 08042702; Agfa HealthCare N.V., Belgium). The following clinical information was collected: age, gender, smoker, body mass index (BMI), ASA score, histopathology of esophageal cancer, general length of hospital stay, length of stay in intensive care unit, and success of treatment. The Ethics Committee of the Medical Faculty at the University of Cologne approved the evaluation. The Institutional Review Board was informed and there were no objections.

### Cost and reimbursement data

Medical and demographic data were merged with economic data using a SAP-based controlling program (eis.TIK; KMS AG Germany). All economic parameters of the patients were analyzed independently of their insurance status by focusing on reimbursement and cost data per inpatient case. Reimbursement and cost allocation per case were based on the German-Diagnosis Related Group (G-DRG) system[11]. For the Case Mix (CM) and financial analysis (including surcharges and discounts), we assumed Base Rates (BR) for the German state North Rhine-Westphalia [12]. No discounting was performed, and all costs and revenues are expressed in Euro (€) based on respective currency rates for the years 2012 to 2016.

### Statistics

The statistical analysis was performed by using SPSS 23 (IBM Corp., Armonk, NY, USA). For descriptive purposes, baseline patient and case characteristics are presented as median and mean. Furthermore, the Wilcoxon-Mann-Whitney-Test was applied to assess the statistical significance of normally distributed length of hospital stay, length of stay at intensive care unit, and CM per case. A P-value <0.05 was considered significant.

## Results

Between January 2012 and December 2016, a total of 660 oncological esophagectomies using an intrathoracic anastomosis were performed in our high-volume center.

**Fig 1:**
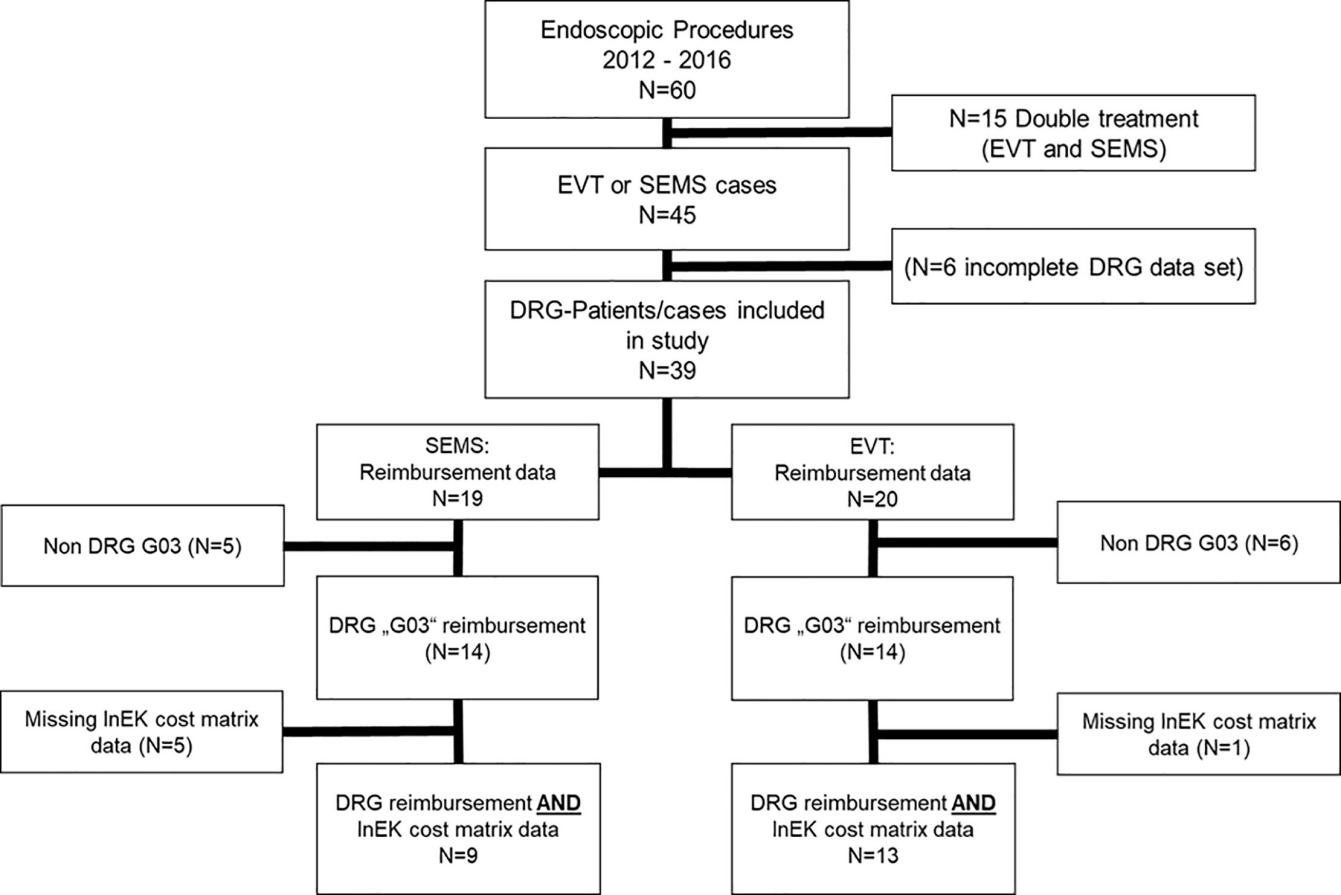
Structure of the economic analysis.

During this time, we identified medical records from 60 patients who received SEMS, EVT, or both for endoscopic management of an anastomotic leak after oncological Ivor Lewis esophageal surgery between January 2012 and December 2016 (Fig 1). To increase transparency between costs related to SEMS or EVT, we excluded fifteen patients because they had received a combination of both treatments (SEMS and EVT). The remaining 45 patients had a median age of 65 years, and a majority of them were male (80%). More than half of the patients were smokers (55.6%). In addition, the median BMI was 26, and the median ASA score was 3. Almost two third of the patients received neoadjuvant treatment (62.2%), including chemo- and or radiation-therapy. The majority of patients suffered from an adenocarcinoma (71.1%), the second largest group suffered from a squamous cell carcinoma (26.7%), and the smallest group of patients suffered from a neuroendocrine tumor (2.2%). Details of the study group are demonstrated in Table 1.

**Table 1:** Patient characteristics and economic performance data.

Variable	Patients with Anastomotic Leak (N = 45)
**Age, median (range)**	65 (43–84)
**Sex**	
**Male, n (%)**	36 (80.0)
**Female, n (%)**	9 (20.0)
**Smoker, n (%)**	25 (55.6)
**BMI, median (range)**	26,00 (17–46)
**ASA score, median (range)**	3 (1–4)
**Neoadjuvant therapy, n (%)**	28 (62.2)
**Histopathology**	
**Adenocarcinoma, n (%)**	32 (71.1)
**Squamous cell carcinoma, n (%)**	12 (26.7)
**Neuroendocrine tumor, n (%)**	1 (2.2)
**Success of Treatment, n (%)**	37 (82.2)
** No. of G-DRG cases / payments** ** (DRG catalogue 2012–2016)**	39
** Case Mix effective (CM eff.)**	515,029
** Case Mix Index (CMI), median**	13.21 (6.52)
**Length of hospital stay, Average (ALOS) / median (range)**	42.85 / 31 (11–296)
**Length of stay in ICU, Average / median (range)**	13.74 / 8 (1–111)

In the cost analysis, another 6 patients had to be excluded due to incomplete cost and / or reimbursement data (e. g., pending regression claims, insufficient quality per cost accounting by InEK algorithm). Finally, 39 patients with complete DRG codes could be analyzed from an economic perspective including N = 19 cases in the SEMS group and N = 20 in the EVT group. For a detailed cost-revenue comparison, an InEK cost-matrix analysis was applied in the SEMS group (N = 9) and the EVT group (N = 13).

The overall case mix (CM) of 39 coded DRGs amounted to about 515 with a case mix index (CMI) of 13.21 (median value 6,52). The average length of hospital stay was 42.85 days (range 11–296 days hospitalization and a median value of 31 days).

In the SEMS group (N = 19), we identified a wide range of different DRG cases (compare Table 2). The highest DRG per case was an Intensive Care Unit (ICU) DRG (A07A) with a CMI of 145.4. The most frequent DRG was G03A as “large interventions on stomach, esophagus and duodenum (…)” with an overall CM of 80.903 (further DRG terms and definitions are provided in Attachment 1). Table 2 shows a total reimbursement for the SEMS group of €1,069,413, and patients had an average length of hospital stay (ALOS) of 7.3 days.

**Table 2:** DRG performance data of SEMS group.

DRG-Code	No. of DRG cases	Ʃ Case Mix effective	Ʃ Length of hospital stay	Ʃ Length of stay, Intensive Care Unit	Ʃ DRG payment	Ʃ Coded extra fees	Ʃ Reimbursement (DRG + extra fees)
**A07A**	1	145	296	111	464,274	2,865	467,139
**A07C**	1	32	73	55	986,55	5,569	104,224
**A09B**	1	23	44	30	68,513	1,945	70,458
**A11A**	1	27	71	57	82,049	7,282	89,331
**A13B**	1	10	20	13	32,474	5,840	38,314
**G03A**	13	81	333	68	247,971	24,760	272,731
**G03C**	1	8	62	3	22,350	4,866	27,215
**Total**	19	326	899	337	1,016,286	53,127	1,069,413

Table 3 shows that the EVT group (N = 20) had the most profitable and most frequently coded DRG G03A with over 71.252 CMI. The total reimbursement in the EVT group was € 674,188 with an average length of stay of over 38 days.

**Table 3:** DRG performance data of EVT group.

DRG- Code	No. of DRG cases	Ʃ Case Mix effective	Ʃ Length of hospital stay	Ʃ Length of stay, Intensive Care Unit	Ʃ DRG payment	Ʃ Coded extra fees	Ʃ Reimbursement (DRG + extra fees)
**901D**	1	2	11	2	7,160	2,766	9,178
**A09C**	1	48	116	31	151,752	7,309	159,061
**A11D**	1	14	48	23	45,297	4,351	49,647
**A13B**	1	16	51	15	33,149	4,322	57,298
**A13D**	1	8	29	11	25,541	3,644	29,184
**G03A**	10	71	359	71	216,241	27,345	255,262
**G03C**	4	20	135	31	60,394	4,351	67,842
**G36C**	1	11	23	15	34,660	12,054	46,714
**Total**	20	189	772	199	574,193	66,141	674,188

### Analysis of DRG G03

In both groups, we identified G03 (three digits DRG/ basic DRG) as the most frequently coded DRG (SEMS = 14; EVT = 14). In the SEMS group, N = 9 of the 14 G03 cases feature complete cost data and show overall payments per case of € 23,549 (average value, total reimbursement of € 211,938), including coded extra fees of € 3,141 per case (average value, in total € 25,126 including € 16,969 extra fees for medications and € 8,156 for implants).

In the EVT group, N = 13 cases had complete financial data and show overall payments per case of € 23,939 (average value, total reimbursement of € 311,213), including coded extra fees of € 4,528 per case (in total € 31,696). The statistical analysis showed no significant differences between both samples (p = 0.845).

**Fig 2:**
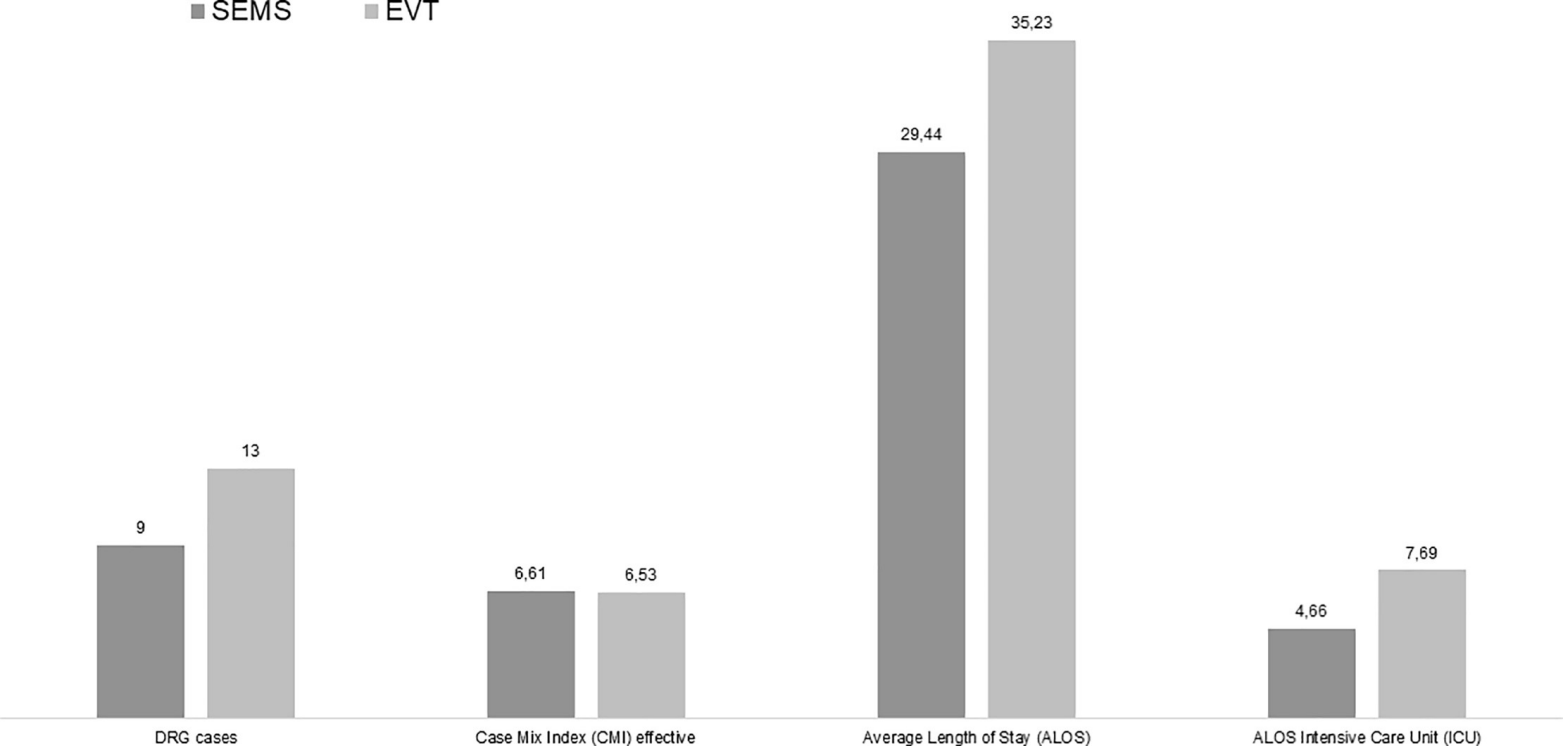
SEMS / EVT comparison of Key Performance Indicators in subgroup DRG G03 (average values).

As shown in Fig 2, the effective CMI was almost alike in both groups. In total, days of hospitalization for the EVT group amounted to 458 days compared to 265 days for the SEMS group (p = 0.647). On average, the length of hospital stay in the EVT group was also higher than that in the SEMS group (EVT average: 35.23, median: 32; SEMS average: 29.44, median: 26) (p = 0.144). Hospitalization days (ALOS) at the ICU were also higher in the EVT group (on average 7.69, median: 8) than in the SEMS group (on average 4.66, median: 3) (p = 0.164). However, based on the statistical analysis, these results for the Key Performance Indicators (KPI) were not statistically significant.

**Fig 3:**
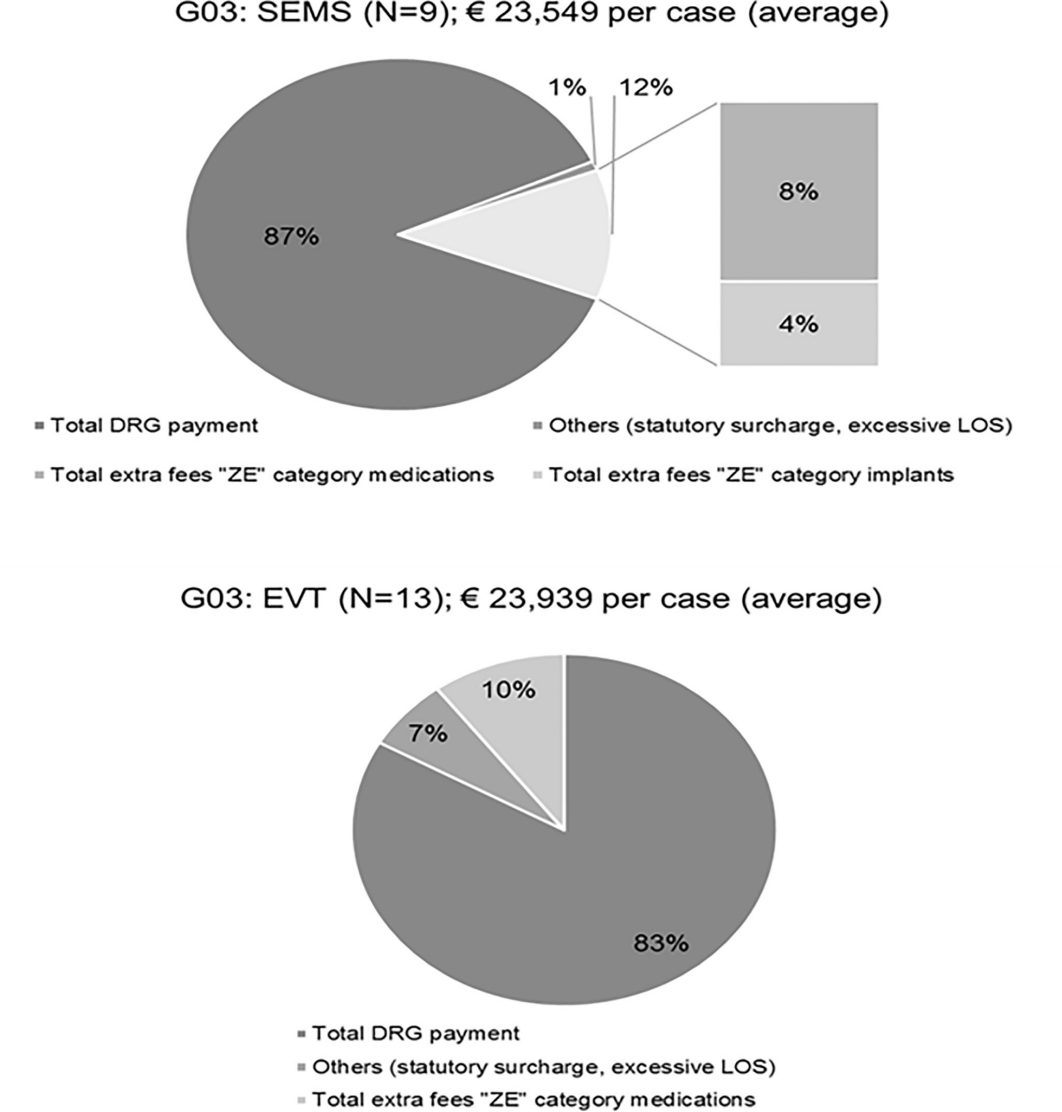
Reimbursement structure in DRG G03: SEMS (N = 9) vs. EVT (N = 13).

As demonstrated in Fig 3, the predominant part of the reimbursement was the flatrate payment DRG for both groups. Differences were identified in (additional) surcharges, for example, due to excessive hospitalization in the EVT group (surcharges for two cases: € 14,775). The total payment of extra fees (“ZE”) were comparable in both groups with 12% of overall reimbursements in the SEMS group and 10% in the EVT group. However, focusing on the extra fee reimbursement category, differences were identified in the type of extra fee. Expensive drugs such as anti-infectives like “anidulafungin” were cost compensated through the DRG catalogue via extra fees (“ZE”) in both groups (cost compensation of medications in SEMS group: € 16,969 and in EVT group: € 31,696). Yet, additional payments for implantations of stents applied only to the SEMS group. Our analysis revealed 10 coded extra fees for implants in the SEMS group with a compensation of € 800 to € 831 per implantation. There was no additional reimbursement for the necessary medical material for EVT in the EVT group.

**Fig 4:**
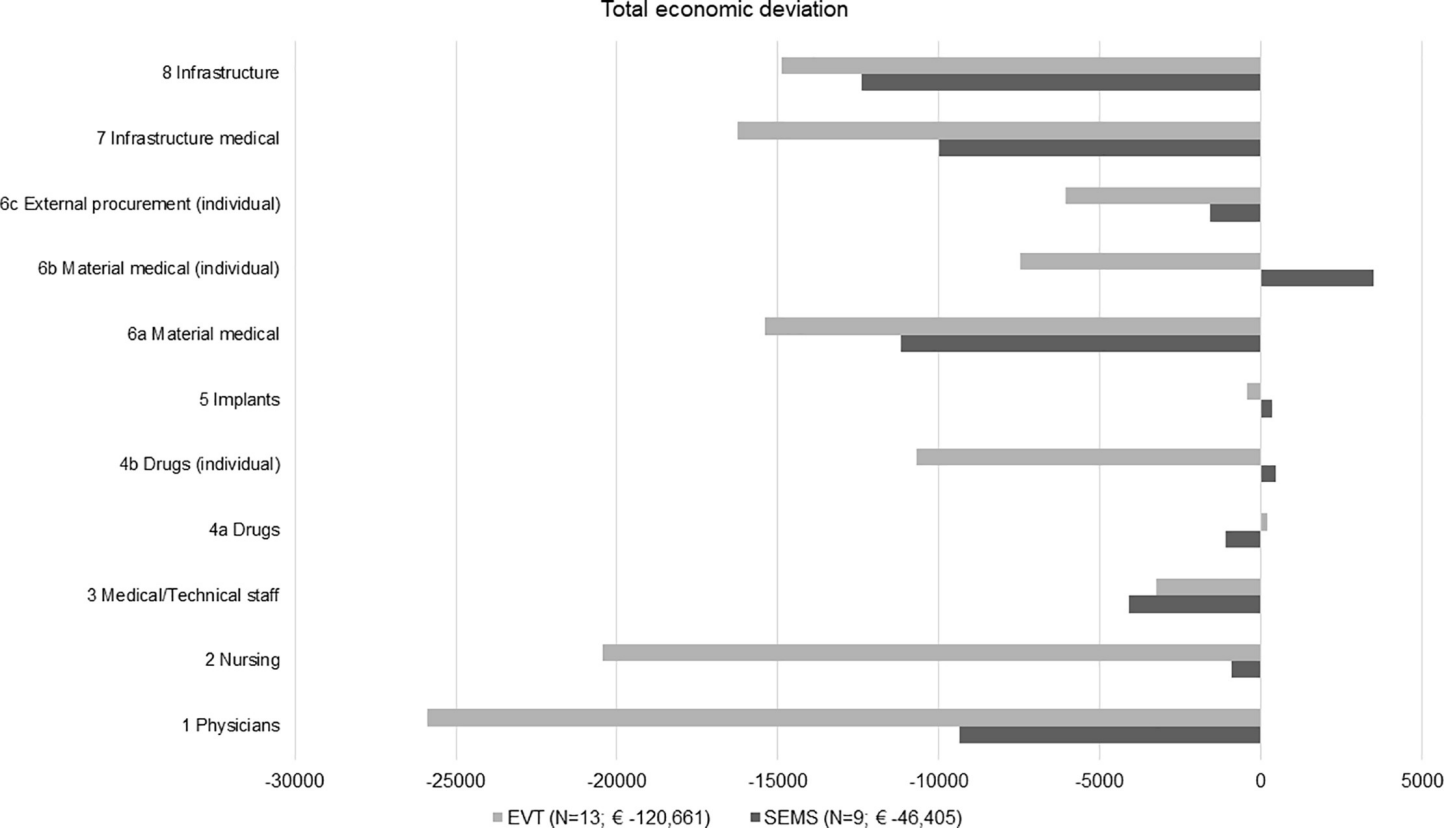
Cost-revenue comparison of InEK cost categories in the SEMS (N = 9) and EVT (N = 13) group.

The total deficit in the SEMS group is high at € – 46,405, and the deficit in the EVT group is almost three times as high at € -120,661. As shown in Fig 4, in both groups and through almost all cost categories (1–8), we made a financial deficit. In the SEMS group, the highest deficit was in cost category “8 Infrastructure” with € -12,399 (total share: 27%) followed by “6a Material, medical” with € -11,175 (total share: 24%). In the EVT group, the biggest cost driver was “1 Physicians” with € -25,878 (total share: 21%) followed by the personnel cost category “2 Nursing” with € - 20,452 (total share: 17%).

In the cost–revenue comparison per case (Tables A and B in S1 Appendix), the InEK cost matrix calculation shows an almost two times higher calculated deficit per case in the EVT group (SEMS: € -5,165 compared to EVT: € -9,282). The predominant cost center in both groups is “1 Ward” with a deficit per case of € - 3,927 followed by “10 Laboratories”with € -1,364 in the SEMS group. In the EVT group, our results show a deficit in this cost center of € - 5,588 per case followed by cost center “8 Endoscopy” with a deficit of € -1,485. Through both groups, the cost-category group analysis has shown high infrastructure deficits per case (SEMS: € -2,490; EVT: € -2,397). The predominant single cost category in the SEMS group was non-medical infrastructure (8 Infrastructure: € -1,378), and in the EVT group, the single cost category personnel costs “1 Physicians” (€ -1,991) was identified as the main cost driver.

## Discussion

Several studies have shown that complications after esophageal surgery are associated with substantial treatment costs and an increased economic burden for the hospitals [4, 5, 13]. In a multivariable analysis, Goense et al. showed that there is a € 4,123 increase in costs per case following anastomotic leaks in the Netherlands (p = 0.008) [5].

From a medical perspective, endoscopic options of SEMS and EVT seem to be equally effective in treating anastomotic leaks after oncological Ivor Lewis esophagectomies [14]. However, a health-economic study that investigates the cost effectiveness of SEMS or EVT treatment for anastomotic leaks from a payer`s perspective has yet to be published.

Since the implementation of G-DRG remuneration in 2003, hospitals in Germany use DRG mechanisms as a cost accounting scheme to optimize their financial results. Whereas lump sum payment varies throughout Europe in terms of cost accounting guidelines, cost collection methods, and data checks, hospitals in Europe increasingly use DRG systems to optimize their performance portfolio [15, 16].

To our knowledge, this is the first health-economic study analyzing reimbursement for endoscopic treatment options of anastomotic leaks with internal case-level-based cost data to support strategic management decisions in hospitals. Our main goal in this analysis was to identify from a hospital management perspective the economic burden, major cost drivers, and financial risks in the endoscopic treatment of anastomotic leaks after oncological Ivor Lewis esophagectomy with SEMS and EVT. Due to financial restrictions, transparency of exact costs for complication management after esophageal surgery becomes more and more important for cancer centers. Hence, a cost comparison of different treatment options, especially of treatment options that seem to be equally efficient from a medical perspective, becomes relevant. Otherwise, medical treatment costs could get out of control and resource allocation would be inefficient from a hospital management perspective.

Several studies have analyzed the costs for endoscopic procedures in the G-DRG-System. In general, costs for endoscopic procedures in Germany are lower than in other European countries [17–19]. Furthermore, higher costs for endoscopic procedures in university hospitals are likely due to referral bias for complex cases as well as emergency interventions [17]. Based on the results of our study, costs for EVT are almost two times higher than those for SEMS treatment, which can be explained by higher medical and nursing staff costs as well as arising material costs. Usually, patients who receive EVT stay longer in the hospital due to changes of endoluminal placed foams; on average, 3 or 4 changes were required until a leak was successfully treated. Medical costs for the implantation of the foam in EVT are not directly compensated and, therefore, lead to a higher deficit compared to that accrued through SEMS treatment.

In both treatment options, extra fees include expenses for costly medications like anti-infectives such as `anidulafungin`or other resource-intensive treatments. However, there is a vital difference in the reimbursement for SEMS treatment as the extra fees for implemented stents make up around one third of all received extra fees. Up to now, there is no extra fee for EVT material, which is why a financial reimbursement for EVT should be discussed. This could ultimately facilitate a comparison between both treatment options from an economical perspective.

In this context, it is important to evaluate DRG factors as major diagnostic categories (MDC) or specific operations and procedure codes (OPS) leading to EVT-DRG with high cost-weight. In the current G-DRG system two codes exist: G35Z (`complex vacuum treatment for diseases and disorders of the digestive system; cost-weight: 11.518) or I98Z (`complex vacuum treatment for diseases and disorders at musculoskeletal system and connective tissue`; cost-weight: 7.816). From our medical point of view and due to medical coding algorithms and conditions in the German DRG system, our EVT group does not benefit from the above mentioned evaluation of DRG factors. To be grouped into DRG G35Z, for example, mandatory requirements for EVT (such as, repeated complex procedures) were not achieved in our groups. Other requirements such as a minimum length of treatment for EVT (8 days), usage of vacuum sealing system as well as MDC06 were easily achieved in our cases. From a DRG system perspective, new definitions, coding conditions, and the implementation of direct cost coverage through extra fees could lead to an adequate economic outcome in the EVT group.

It should also be considered that due to lower complication rates following esophageal surgery in high-volume centers compared to low-volume centers, a centralization of these complex oncological surgeries should be favored. From a payer`s perspective, this would likewise help to reduce general treatment costs resulting from potential postoperative complications.

To assess the costs and reimbursement mechanisms at our high volume-center, we have implemented a profit-center calculation system according to the InEK cost-accounting approach, comparing hospital reimbursement data with internal costs per case. Determining costs and payments on case- and patient-level, we were able to calculate profit margins and loss per case. The results clearly indicate that novel reimbursement structures for SEMS treatment and especially for EVT procedures are needed and should be considered for a future DRG system. Due to calculation delays in the DRG reimbursement system, suppliers and for-profit organizations should also evaluate cost adjustments for those cost-intensive operative procedures in high volume centers.

Our analysis has several limitations such as a primary focus on direct cost not considering costs from the societal perspective. Further economic evaluations should also integrate outpatient treatment options and related costs in leak treatment. Furthermore, our findings are relevant for all countries using the DRG-system, but they need to be applied in each country from their specific perspective.

## Conclusion

Our case-level based profit center analysis demonstrates that endoscopic management of anastomotic leaks in the upper gastrointestinal tract with SEMS and EVT is not cost covering from a hospital management perspective. EVT inpatient therapy is twice as costly as SEMS treatment. Effective cost-revenue controlling is crucial to avoid major economic risk for the hospital, and an improved reimbursement system of these endoscopic therapies is needed.

## Supporting information

S1 Appendix Table A. Profit margin analysis per case of DRG G03 in SEMS group (N = 9) (all data in €). Table B. Profit margin analysis per case of DRG G03 in EVT group (N = 13) (all data in €).
(DOCX)

## Abbrevations

ALOS: Average Length of Stay
CM: Case Mix
CMI: Case Mix Index
DRG: Diagnosis Related Group
EVT: Endoscopic Vacuum Therapy
ICU: Intensive Care Unit
INek: Institute for Remuneration System in Hospitals
MDC: Major Diagnostic Categories
OPS: Operations and Procedures Codes
SAP: “S”ystems, “A”pplications & “P”roducts in Data Processing
SEMS: Self-expanding Metal Stent
ZE: Zusatzentgelt („extra fees”)
