# Supplementary material for: The economic burden of endoscopic treatment for anastomotic leaks following oncological Ivor Lewis esophagectomy
Source: PLoS One. 2019 Aug 28;14(8):e0221406. doi: 10.1371/journal.pone.0221406 (PMC6713440; doi:10.1371/journal.pone.0221406)
Supplement: S1 Appendix — Table A. Profit margin analysis per case of DRG G03 in SEMS group (N = 9) (all data in €). Table B. Profit margin analysis per case of DRG G03 in EVT group (N = 13) (all data in €). (DOCX) [file pone.0221406.s001.docx]

**S1 Appendix**

| **InEK matrix**  **(deviation per case)** | **Personnel costs** | | | **Material costs** | | | | | | **Infrastructure costs** | |  |
| --- | --- | --- | --- | --- | --- | --- | --- | --- | --- | --- | --- | --- |
| **Cost category groups**  **Cost-center groups** | **1 Physicians** | **2 Nursing staff** | **3 Medical/Technical staff** | **4a Drugs** | **4b Drugs (individual)** | **5 Implants** | **6a Medical material** | **6b Medical material (individual)** | **6c External procurement (individual)** | **7 Infrastructure (medical)** | **8 Infrastructure** | **Total** |
| **1 Ward** | -472 | -814 | 40 | -224 | -1,189 | 0 | -197 | 13 | 0 | -516 | -569 | -3,927 |
| **2 Intensive Care** | 195 | 708 | 20 | 111 | 1,131 | 0 | -60 | 11 | 0 | -33 | 98 | 2,181 |
| **3 Dialysis** | 0 | 0 | 0 | 0 | 0 | 0 | 0 | 0 | 0 | 0 | 0 | 0 |
| **4 Operating rooms** | 243 | 0 | 154 | 5 | -6 | 13 | -524 | 679 | 0 | -282 | -161 | 121 |
| **5 Anesthesia** | -157 | 0 | -275 | -21 | 3 | 0 | -47 | 1 | 0 | -140 | -242 | -879 |
| **6 Maternity unit** | 0 | 0 | 0 | 0 | 0 | 0 | 0 | 0 | 0 | 0 | 0 | 0 |
| **7 Cardiology** | 2 | 0 | 2 | 0 | 0 | 1 | 0 | 1 | 0 | 1 | 1 | 8 |
| **8 Endoscopy** | -347 | 0 | -146 | -3 | 0 | 23 | -72 | -289 | 0 | -102 | -205 | -1,141 |
| **9 Radiology** | -40 | 0 | -49 | -2 | 0 | 1 | -14 | 18 | 0 | -34 | -18 | -138 |
| **10 Laboratories** | -524 | 0 | -416 | 5 | 111 | 0 | -333 | 165 | 0 | -17 | -356 | -1,364 |
| **11 Further diagnostics** | 59 | 3 | 213 | 5 | 0 | 0 | 6 | -212 | -177 | 11 | 74 | -18 |
| **Total** | **-1,040** | **-103** | **-457** | **-122** | **50** | **38** | **-1,242** | **388** | **-177** | **-1112** | **-1,378** | **-5,156** |

**Table A. Profit margin analysis per case of DRG G03 in SEMS group (N=9) (all data in €).**

**Table B. Profit margin analysis per case of DRG G03 in EVT group (N=13) (all data in €).**

| **InEK matrix**  **(deviation per case)** | **Personnel costs** | | | **Material costs** | | | | | | **Infrastructure costs** | |  |
| --- | --- | --- | --- | --- | --- | --- | --- | --- | --- | --- | --- | --- |
| **Cost category groups**  **Cost-center groups** | **1 Physicians** | **2 Nursing staff** | **3 Medical/Technical staff** | **4a Drugs** | **4b Drugs (individual)** | **5 Implants** | **6a Medical material** | **6b Medical material (individual)** | **6c External procurement (individual)** | **7 Infrastructure (medical)** | **8 Infrastructure** | **Total** |
| **1 Ward** | -782 | -1,342 | 51 | -51 | -1,917 | 0 | -172 | -290 | 0 | -539 | -546 | -5,588 |
| **2 Intensive Care** | -607 | -237 | 1 | 70 | 1,081 | 0 | -264 | -59 | 0 | -291 | -256 | -562 |
| **3 Dialysis** | 0 | 0 | 0 | 0 | 0 | 0 | 0 | 0 | 0 | 0 | 0 | 0 |
| **4 Operating rooms** | 182 | 0 | 12 | 0 | 17 | 14 | -440 | 66 | 0 | -238 | -100 | -489 |
| **5 Anesthesia** | -43 | 0 | -81 | -7 | 4 | 0 | -48 | 1 | 0 | -29 | 50 | -153 |
| **6 Maternity unit** | 0 | 0 | 0 | 0 | 0 | 0 | 0 | 0 | 0 | 0 | 0 | 0 |
| **7 Cardiology** | 2 | 0 | 2 | 0 | 0 | 0 | 0 | 1 | 0 | 1 | 1 | 7 |
| **8 Endoscopy** | -514 | 0 | -177 | -2 | -1 | -49 | -70 | -343 | 0 | -104 | -227 | -1,485 |
| **9 Radiology** | -105 | 0 | -71 | -1 | 0 | 0 | -28 | -8 | 0 | -79 | -31 | -323 |
| **10 Laboratories** | -147 | 0 | -188 | 3 | -6 | 0 | -168 | 180 | 0 | 16 | -104 | -414 |
| **11 Further diagnostics** | 25 | 6 | 201 | 4 | 0 | 0 | 4 | -124 | -468 | 11 | 67 | -275 |
| **Total** | **-1,991** | **-1,573** | **-250** | **15** | **-822** | **-34** | **-1,186** | **-576** | **-468** | **-1,252** | **-1,146** | **-9,282** |
